# Regional Variations of Insulin Secretion and Insulin Sensitivity in Japanese Participants With Normal Glucose Tolerance

**DOI:** 10.3389/fnut.2021.632422

**Published:** 2021-03-22

**Authors:** Kiriko Watanabe, Moritake Higa, Yoshimasa Hasegawa, Akihiro Kudo, Richard C. Allsopp, Bradley J. Willcox, Donald C. Willcox, Masataka Sata, Hiroaki Masuzaki, Michio Shimabukuro

**Affiliations:** ^1^Department of Diabetes, Endocrinology and Metabolism, School of Medicine, Fukushima Medical University, Fukushima, Japan; ^2^Department of Diabetes and Life-Style Related Disease Center, Tomishiro Central Hospital, Okinawa, Japan; ^3^Department of Cardiovascular Medicine, Institute of Biomedical Sciences, Tokushima University Graduate School, Tokushima, Japan; ^4^Department of Internal Medicine, Ohara General Hospital, Fukushima, Japan; ^5^Institute for Biogenesis Research, University of Hawaii, Honolulu, HI, United States; ^6^Pacific Health Research and Education Institute, Honolulu, HI, United States; ^7^Department of Geriatric Medicine, John A. Burns School of Medicine, University of Hawaii, Honolulu, HI, United States; ^8^Department of Human Welfare, Okinawa International University, Okinawa, Japan; ^9^Department of Cardiovascular Medicine, Institute of Biomedical Sciences, Tokushima University Graduate School, Tokushima, Japan; ^10^Division of Endocrinology, Diabetes and Metabolism, Hematology, Rheumatology (Second Department of Internal Medicine), Graduate School of Medicine, University of the Ryukyus, Okinawa, Japan

**Keywords:** insulin secretion, insulin sensitivity, obesity, nutrients, fatty acids

## Abstract

**Purpose:** Regional differences in dietary patterns in Asian countries might affect the balance of insulin response and sensitivity. However, this notion is yet to be validated. To clarify the regional differences in the insulin response and sensitivity and their relationship to nutrients, we compared the insulin secretory response during an oral glucose tolerance test in Japanese participants.

**Methods:** This observational retrospective cohort study analyzed the data from participants with normal glucose tolerance (NGT) from four distinct areas of Japan with regard to the food environment: Fukushima, Nagano, Tokushima, and Okinawa based on data available in the Japanese National Health Insurance database.

**Results:** Although the glucose levels were comparable among the four regions, the insulin responses were significantly different among the regions. This difference was observed even within the same BMI category. The plot between the insulin sensitivity index (Matsuda index) and insulin_AUC_/glucose_AUC_ or the insulinogenic index showed hyperbolic relationships with variations in regions. The indices of insulin secretion correlated positively with fat intake and negatively with the intake of fish, carbohydrate calories, and dietary fiber.

**Conclusions:** We found that significant regional differences in insulin response and insulin sensitivity in Japanese participants and that nutritional factors may be linked to these differences independently of body size/adiposity. Insulin response and insulin sensitivity can vary among adult individuals, even within the same race and the same country, and are likely affected by environmental/lifestyle factors as well as genetic traits.

## Introduction

Diabetes mellitus is a metabolic disease characterized by hyperglycemia resulting from deficiency in insulin secretory capacity, insulin action or both (1). Kodama et al. compared the racial/ethnic differences for an optimal balance between insulin secretion and insulin sensitivity among Africans, Caucasians, and East Asians (2), and found that East Asians had better insulin sensitivity and lower insulin secretory capacity than Africans and Caucasians. However, a change in the living environment, such as through immigration, affects the balance between insulin secretion and insulin sensitivity even in individuals with the same racial/ethnic features. Fujimoto et al. reported that, after adjusting for the body mass index (BMI), the insulin response of Japanese-Americans was higher than that of native Japanese, suggesting that factors other than body size/adiposity play a distinct role in mediating insulin secretion (3).

Nutrients are plausible candidates as factors that mediate an altered insulin response (4). Immigration from Asia to Europe or the USA might change cultural dietary patterns, such as the intake of staple food, meat, and fish, with resultant changes in the balance among protein, fat, and carbohydrate intake (5). The elevation of plasma concentrations of free fatty acid (FFA) stimulated insulin secretion during the euglycemic or hyperglycemic clamp state (6). McGarry et al. confirmed that, in the insulin secretory pathway, a fatty acid-derived lipid moiety plays a pivotal role that is common to the action of a wide variety of secretagogues: highly saturated FFA (SFA), but not n-3 polyunsaturated fatty acids (PUFA), overstimulates the insulin response independent of the glucose levels (7). The Western diet is characterized by a high intake of meat and reduced intake of fish, which results in high SFA and low n-3 PUFA plasma concentrations (5). Taken together, the westernized dietary pattern could be closely related to an altered insulin response and insulin sensitivity through the hazardous effects of SFA, which is considered to be a form of “lipotoxicity” (8, 9). Regional differences in dietary patterns in Asian countries might affect the balance of insulin response and sensitivity. However, this notion is yet to be validated.

To clarify the regional differences in the insulin response and sensitivity and their relationship to nutrients, we compared the insulin secretory response during an oral glucose tolerance test in Japanese participants. We selected participants with normal glucose tolerance (NGT) to minimize the effects of personal glucose intolerance and chose four distinct areas of Japan with regard to the food environment: Fukushima, Nagano, Tokushima, and Okinawa (Supplementary Figure 1).

## Materials and Methods

### Study Design and Study Participants

This observational retrospective cohort study included an analysis in the Kokuho Database (KDB) which includes data form Japanese National Health Insurance and the data obtained during annual specific health checkups according to “The Specific Health Check and Guidance in Japan” (10, 11). Of the 47 regions (prefectures) in Japan, we selected four distinct regions based on the food environment (12) and structure of non-communicable diseases (13): Fukushima, Nagano, Tokushima, and Okinawa. From the overall list of 4,393 candidates who had undergone an oral 75-g glucose tolerance test (OGTT) between April 2005 and December 2016 in the KDB of four regions, we randomly selected study participants with the assumption that the participants were representative of candidates from each region (Supplementary Figure 2).

To obtain representative data for the daily intake of nutrients and food in four regions, we used data from the National Health and Nutrition Survey (NHNS), a series of nationally representative cross-sectional nutrition surveys conducted by local public health centers (12). The personalized data of the participants in the NHNS database were not available for their privacy policy, therefore, the researchers could only report aggregate data. However, since the nutritional samples in NHNS had been extracted and collected with a statistically rigorous manner to mimic real distributions in 47 regions (prefectures), we considered these as representative values for each region.

The study protocol was approved by the Fukushima Medical University Ethics Committee (approval number 30168). An opt-out notice for tacit consent was posted to publish the research information and ensure the opportunity for refusal. This study was conducted in accordance with the Ethical Guidelines for Medical and Health Research Involving Human Subjects enacted by the MHLW of Japan.

### Anthropometric and Biochemical Measurements

The procedures for the anthropometric and biochemical measurements have been described previously (11). Briefly, trained staff measured height, body weight, waist circumference, and systolic and diastolic blood pressure (SBP and DBP) using a standard sphygmomanometer or an automated device on the right arm after resting for 5 min in a seated position. The participants reported their age, sex, smoking and alcohol intake, medications, and history of cardiovascular diseases and cerebrovascular disease in the questionnaire. Blood samples were collected after overnight fasting >12 h. Centrifuged samples were analyzed using an automatic clinical chemistry analyzer within 3 h of sample collection. All blood samples were analyzed at local laboratories by using the standard methods for laboratory tests recommended by the Japan Society of Clinical Chemistry. The analyzed factors were fasting plasma glucose (FPG), low density lipoprotein (LDL)-cholesterol, high density lipoprotein (HDL)-cholesterol, and triglyceride. Hypertension was defined as SBP ≥ 140 mmHg, DBP ≥ 90 mmHg, or use of antihypertensive medication. Dyslipidemia was defined as LDL-cholesterol 140 ≥ mg/dL, HDL- cholesterol ≤ 40 mg/dL, TG ≥ 150 mg/dL, or use of anti-dyslipidemic drugs. If necessary, the total cholesterol was calculated by the Friedewald's method (14), and participants whose TG was ≥400 mg/dL were excluded from the analysis.

### Indices of Dietary Factors

Descriptive reports of the NHNS data, including health status, food and nutrient intakes, and lifestyles of Japanese civilians, are available (15). Household members were randomly selected from 475 out of ~1,00,000 nationwide census units in Japan. The average indices of dietary factors are available publicly in each region. Data on the dietary intake were collected using a 1-day semi-weighted household dietary record. Participants recorded food consumption at each household for a typical day. Trained fieldworkers, mainly registered dietitians, visited each household and checked the completeness of the data recording forms and, if necessary, confirmed portion sizes by using commercially available food models or food booklets, and corrected any missing and/or illogical information. In accordance with a survey manual of the NHNS, the trained fieldworkers converted these estimates of portion sizes or quantity of foods that were recorded into the weights of the foods and coded each food item according to the NHNS food number lists to calculate the energy and nutrient intake based on the record of household food consumption. The energy intake was calculated by multiplying the intake amounts of protein, lipids, and carbohydrates by applying the Atwater factors. Energy and nutrient intakes from food items with these values that are listed in the Standard Tables of Food Composition in Japan were calculated accordingly (16).

### OGTT and Definition of Glucose Tolerance

After overnight fasting (>12 h), venous blood samples were collected before and after 30, 60, and 120 min of the ingestion of 75 g glucose (Trelan-G75; AY Pharmaceuticals Co., Ltd., Tokyo, Japan). Plasma samples for the assessment of insulin were collected in EDTA tubes on ice, separated after centrifugation (~3,000 Åg for 15 min at 4°C), and stored at −80°C until the assays were performed. Insulin was assayed routinely using a commercially available electrochemiluminescence immunoassay (CLEIA). The status of glucose tolerance was defined according to the WHO 2006 criteria (13): normal glucose tolerance (NGT; fasting plasma glucose <110 mg/dL and 2 h post-load plasma glucose <140 mg/dL), impaired glucose tolerance (IGT; fasting plasma glucose levels of 110–125 md/dL and/or 2 h post-load glucose levels of 140–199 mg/dL) and type 2 diabetes (fasting plasma glucose ≥ 126 mg/dL and/or 2 h post-load glucose ≥ 126 mg/dL).

### Insulin Secretion and Insulin Sensitivity

The OGTT-based indices of insulin secretion and insulin sensitivity were extracted from the literature. Indices of insulin secretion included insulin_0_, insulin_AUC_, insulin_AUC_/glucose_AUC_, homeostatic model assessment estimates of β-cell function (HOMA-β), and the insulinogenic index. Insulin secretion included homeostatic model assessment estimates of insulin resistance (HOMA-IR), insulin sensitivity index (Matsuda index), quantitative insulin sensitivity check index (QUICKI), and the overall index included insulin_120_ and disposition index (1, 17–19). Kahn et al. (1) first reported that the relationship between insulin sensitivity and β-cell function (acute insulin response, AIR_glucose_) in human participants with NGT showed a hyperbolic curve in relation to the results of the intravenous glucose tolerance test (IVGTT) based on the minimum model of glucose kinetics developed by Bergman et al. (20). We used the indices based on OGTT to measure the hyperbolic relationship between the insulin sensitivity index (Matsuda index) (18) and β-cell function (insulinogenic index and insulin_AUC_/glucose_AUC_) (17, 21).

### Statistical Analysis

Categorical variables are expressed as frequencies with percentages, and continuous variables are expressed as means with standard deviation (SD) or medians with interquartile ranges (IQRs). Intergroup comparisons of non-parametric variables were undertaken using the Kruskal–Wallis test, followed by Dunn's multiple comparisons test. Categorical variables are shown as percentages and were analyzed using Fisher's exact test. We used the values of participants from Tokushima as a control because the indices of insulin secretion and sensitivity were different from those of the other three regions. The sex-stratified Pearson product correlation coefficients were calculated for the correlation between indices of insulin response and sensitivity and that of nutrients for participants from the four regions. We performed a multiple regression analysis to estimate insulin_AUC_/glucose_AUC_. Although OGTT data sample size was 2,259, variables on the National nutritional survey were not available for each of 2,259 due to restrictions by a protection law on personal information. Instead, a total of 8 samples were obtained for the mean values of men and women in 4 prefectures. Given limited numbers in our samples (*n* = 8), overfitting multiple regression models could produce misleading coefficients, R-squared and *p*-values. We therefore adopted two independent variables in the model: one dietary composition and the other variable such as age, sex and BMI. Statistical analyses were performed using Prism version 8 or SPSS statistics version 27.0 for Windows (SPSS, IBM Corp, Armonk, NY, USA). All *P*-values were two-sided, and *P* < 0.1 was considered statistically significant.

## Results

Of the 4,393 patients in the four regions who underwent an OGTT, we selected 2,259 (men, 1,190; women, 1,069) participants with NGT for inclusion in this study analysis (Supplementary Figure 2). The general characteristics of the participants are shown in Supplementary Figure 3. The median age, BMI, and waist circumference of men and women, respectively, were 56.0 and 58.0 (47.0–63.0 and 50.0–63.0) years, 25.5 and 25.9 (23.8–27.3 and 24.0–27.8) kg/m^2^, and 89.2 and 90.0 (86.0–94.0 and 84.9–94.0) cm. Among the men, the age, BMI, waist circumference, SBP, DBP, and triglycerides, except LDL-C and HDL-C were significantly different among the four regions. For women, all items evaluated showed significant differences among the regions.

**Figure 1:**
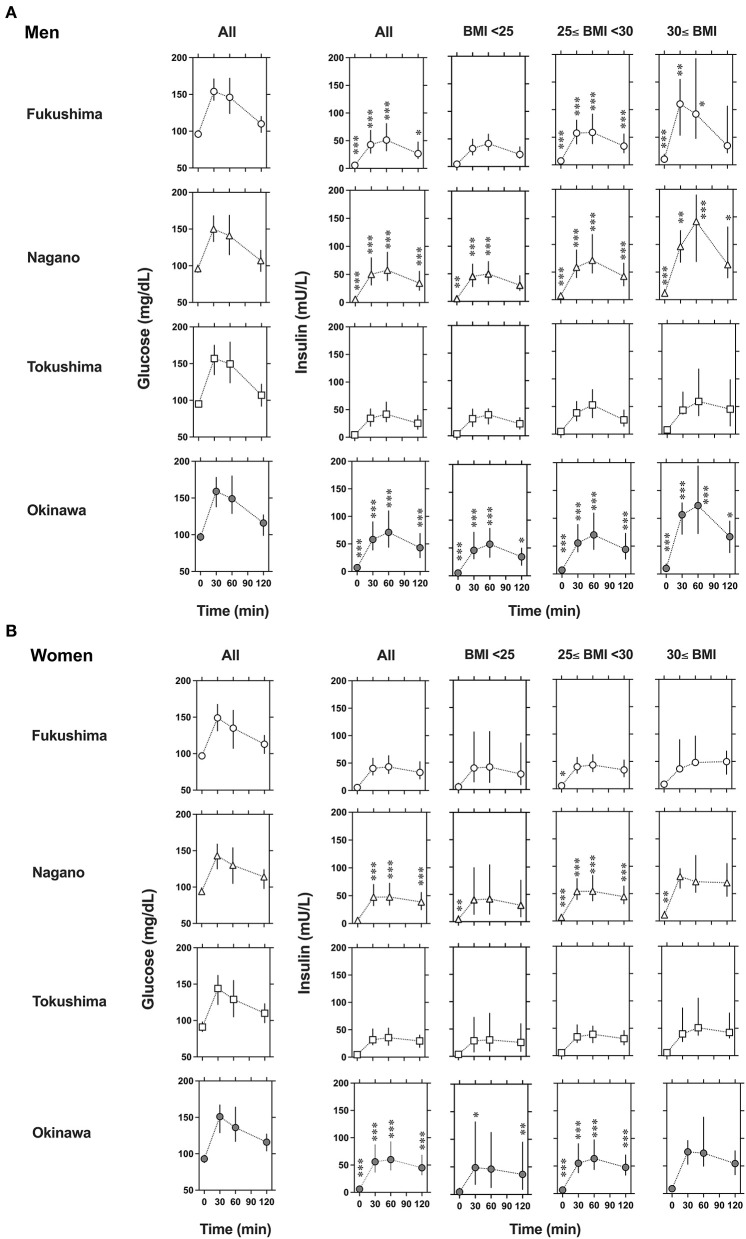
Changes in glucose and insulin levels in men **(A)** and women **(B)** during the 75-g oral glucose tolerance test. Glucose levels are shown in left panels and insulin levels are shown in right panels for all or stratified categories across three body mass index (BMI), BMI <25, 25 ≤ BMI <30, and BMI ≥ 30. The participants were randomly selected from the National Health Insurance database in Fukushima (°), Nagano (Δ), Tokushima (□) and Okinawa (•), Japan. Data are presented as median (25–75th percentile). To compare deference between the regions at each point, *P*-values were obtained by Kruskal–Wallis test, followed by Dunn's multiple comparisons test using Tokushima as a control. *P < 0.1, **P < 0.05, and ***P < 0.01 vs. Tokushima.

The glucose and insulin levels during the OGTT are shown in Supplementary Figure 4 and Figure 1A (men) and Figure 1B (women). The glucose levels were comparable among the four regions in the groups of both men and women. For the insulin responses in men, Fukushima ≈ Nagano ≈ Okinawa > Tokushima in all, 25 ≤ BMI <30 and BMI ≥ 30 and Nagano ≈ Okinawa > Fukushima ≈ Tokushima in BMI <25 (Supplementary Figure 4 and Figure 1A). For the insulin response in women, Nagano ≈ Okinawa > Fukushima ≈ Tokushima in all and BMI <25 and 25 ≤ BMI <30 (Supplementary Figure 4 and Figure 1B).

The indices of insulin secretory capacity and insulin sensitivity are shown in Table 1A (men) and Table 1B (women). Indices of the insulin secretory capacity, such as insulin_0_, insulin_120_, insulin_AUC_, HOMA-β, and the insulinogenic index were larger overall in men and women in Fukushima, Nagano, and Okinawa than in those from Tokushima. The insulin_AUC_/glucose_AUC_ ratio during the OGTT in men and women in the three BMI categories are shown in Supplementary Figure 5. Insulin sensitivity indices, such as HOMA-IR, Matsuda index, QUICKI, and disposition index, were better in participants from Tokushima than from those in the other regions (Tables 1A, 1B).

**Table 1A:** Indices of insulin secretion and insulin sensitivity in men.

	**Insulin 0**	**Insulin 120**	**Insulin_**AUC**_**	**Insulin_**AUC**_/Glucose_**AUC**_**	**HOMA-β**	**Insulinogenic index**	**HOMA-IR**	**insulin sensitivity index (Matsuda index)**	**QUICKI**	**Disposition index**
**Overall**										
Fukushima	5.5 (4.2–7.9)	26.7 (17.7–47.5)	4,631 (2,976–7,278)	0.187 (0.307–0.459)	60.6 (43.6–89.7)	0.73 (0.37–1.13)	1.29 (0.93–1.91)	6.83 (4.42–9.79)	0.368 (0.346–0.389)	74.9 (54.9–112.3)
Nagano	5.9 (4.2–8.0)	34.5 (21.8–55.6)	5,483 (3,831–8,076)	0.254 (0.357–0.521)	67.2 (46.4–94.7)	0.82 (0.48–1.42)	1.41 (0.97–1.92)	6.04 (4.35–8.47)	0.363 (0.346–0.386)	87.2 (55.3–144.9)
Tokushima	4.4 (3.0–6.2)	25.4 (14.5–39.7)	3,748 (2,573–5,805)	0.178 (0.231–0.296)	54.9 (38.7–74.0)	0.53 (0.34–0.88)	0.81 (0.55–1.15)	10.0 (8.23–15.1)	0.397 (0.375–0.426)	92.9 (54.3–197.0)
Okinawa	7.0 (5.0–10.0)	43.0 (25.0–69.0)	6,473 (4,586–9,489)	0.281 (0.403–0.594)	73.2 (51.6–111.9)	0.87 (0.45–1.58)	1.57 (1.11–2.33)	5.20 (3.56–7.24)	0.357 (0.337–0.378)	75.8 (44.6–126.8)
*P* for trend	<0.001	<0.001	<0.001	<0.001	<0.001	<0.001	<0.001	<0.001	<0.001	<0.001
Fukushima*	<0.001	0.070	0.030	<0.001	ns	0.100	<0.001	<0.001	<0.001	ns
Nagano*	<0.001	<0.001	<0.001	<0.001	<0.001	<0.001	<0.001	<0.001	<0.001	ns
Okinawa*	<0.001	<0.001	<0.001	<0.001	<0.001	<0.001	<0.001	<0.001	<0.001	0.009
**BMI** **<** **25**										
Fukushima	4.3 (3.2–5.8)	22.0 (19.8–46.6)	3,421 (2,431–5,457)	0.240 (0.164–0.342)	51.8 (33.2–66.5)	0.54 (0.31–1.06)	1.07 (0.76–1.34)	8.50 (6.10–11.44)	0.380 (0.366–0.402)	77.8 (55.8–135.1)
Nagano	5.0 (3.5–6.7)	29.3 (19.8–46.6)	4,619 (3,293–6,854)	0.309 (0.215–0.459)	55.4 (39.8–80.1)	0.73 (0.41–1.26)	1.16 (0.83–1.58)	7.42 (5.30–10.26)	0.374 (0.356–0.396)	81.9 (53.9–145.0)
Tokushima	3.4 (2.6–5.1)	21.9 (12.2–32.9)	3,212 (2,198–4,531)	0.251 (0.181–0.300)	54.9 (42.9–71.1)	0.54 (0.38–0.93)	0.95 (0.66–1.22)	9.64 (7.64–11.56)	0.389 (0.373–0.416)	87.6 (60.7–156.5)
Okinawa	5.0 (4.1–6.4)	34.1 (18.9–49.8)	5,273 (3,726–7,403)	0.340 (0.228–0.469)	57.0 (41.7–79.8)	0.70 (0.40–1.40)	1.21 (0.91–1.56)	6.62 (5.16–8.74)	0.371 (0.357–0.390)	88.4 (48.2–142.5)
*P* for trend	0.003	0.005	<0.001	<0.001	ns	0.050	0.002	<0.001	<0.001	ns
Fukushima*	ns	ns	ns	ns	ns	ns	ns	ns	ns	ns
Nagano*	0.030	ns	<0.001	<0.001	ns	ns	0.009	<0.001	0.004	ns
Okinawa*	0.004	0.070	<0.001	<0.001	ns	ns	0.001	<0.001	<0.001	ns
**25** **≤** **BMI** **<** **30**										
Fukushima	7.1 (4.9–9.4)	33.9 (21.8–55.8)	5,792 (3,702–7,608)	0.362 (0.232–0.474)	69.3 (48.6–91.8)	0.75 (0.52–1.15)	1.54 (1.04–2.13)	5.62 (4.10–8.05)	0.358 (0.341–0.381)	69.4 (53.8–104.4)
Nagano	5.0 (3.5–6.7)	42.4 (25.2–66.0)	6,248 (4,586–8,773)	0.399 (0.291–0.570)	75.8 (58.2–103.2)	0.95 (0.59–1.46)	1.63 (1.20–2.14)	5.22 (3.58–6.86)	0.355 (0.340–0.372)	88.5 (56.8–143.9)
Tokushima	4.8 (3.8–6.5)	25.8 (14.2–43.6)	3,221 (1,920–9,671)	0.224 (0.166–0.285)	51.4 (36.0–69.4)	0.51 (0.32–0.88)	0.74 (0.51–1.20)	11.56 (7.94–16.85)	0.404 (0.372–0.431)	97.8 (54.1–249.4)
Okinawa	7.0 (4.9–9.5)	44.2 (26.8–73.0)	6,494 (4,497–9,671)	0.393 (0.278–0.586)	75.8 (54.8–103.7)	0.81 (0.45–1.56)	1.68 (1.18–2.34)	4.98 (3.47–6.93)	0.353 (0.336–0.374)	67.7 (42.4–112.8)
*P* for trend	<0.001	<0.001	<0.001	<0.001	<0.001	<0.001	<0.001	<0.001	<0.001	<0.001
Fukushima*	<0.001	0.009	<0.001	<0.001	0.005	0.100	<0.001	<0.001	<0.001	ns
Nagano*	<0.001	<0.001	<0.001	<0.001	<0.001	<0.001	<0.001	<0.001	<0.001	ns
Okinawa*	<0.001	<0.001	<0.001	<0.001	<0.001	<0.001	<0.001	<0.001	<0.001	0.010
**30** **≤** **BMI**										
Fukushima	7.6 (3.9–9.7)	45.3 (14.8–98.6)	8,709 (5,254–17,224)	0.620 (0.321–1.043)	147.0 (96.7–223.5)	1.77 (0.78–2.72)	2.27 (1.80–4.47)	4.30 (1.93–5.61)	0.337 (0.209–0.349)	81.7 (68.7–154.4)
Nagano	6.8 (5.3–9.9)	44.4 (31.5–63.8)	13,150 (7,037–16,590)	0.708 (0.430–1.068)	121.0 (94.4–205.6)	1.48 (0.96–1.89)	2.71 (1.86–4.67)	2.96 (1.97–5.36)	0.329 (0.306–0.348)	62.3 (43.3–163.5)
Tokushima	5.3 (3.8–6.8)	31.0 (19.7–45.8)	4,500 (2,206–5,131)	0.334 (0.194–0.381)	81.8 (59.6–126.6)	0.76 (0.63–0.96)	0.52 (0.34–0.96)	9.99 (8.75–23.74)	0.429 (0.386–0.476)	178.9 (112.7–320.5)
Okinawa	7.0 (4.9–9.5)	44.2 (26.8–73.0)	11,714 (7,920–14,720)	0.627 (0.495–0.874)	134.4 (87.7–202.0)	1.41 (0.94–2.16)	2.53 (1.90–4.15)	3.46 (2.15–4.18)	0.332 (0.310–0.347)	79.3 (48.3–151.0)
*P* for trend	0.006	0.060	0.004	0.007	ns	ns	0.004	0.003	0.004	ns
Fukushima*	0.007	ns	0.050	0.050	ns	0.080	0.007	0.020	0.007	ns
Nagano*	0.002	0.050	0.002	0.003	ns	0.080	0.001	0.001	0.001	0.080
Okinawa*	0.002	0.060	0.002	0.003	ns	0.060	0.001	<0.001	0.001	0.070

Data are presented as median (25–75th percentile). BMI, body mass index; AUC, area under the curve; HOMA-β, homeostatic model assessment estimates of β-cell function; HOMA-IR, homeostatic model assessment estimates of insulin resistance; QUICKI, quantitative insulin sensitivity check index; ns, not significant. P-values were obtained by Kruskal–Wallis test, followed by Dunn's multiple comparisons test. P < 0.1 considered statistically significant. * vs. Tokushima.

**Table 1B:** Indices of insulin secretion and insulin sensitivity in women.

	**Insulin 0**	**Insulin 120**	**Insulin_**AUC**_**	**Insulin_**AUC**_/Glucose_**AUC**_**	**HOMA-β**	**Insulinogenic index**	**HOMA-IR**	**Insulin sensitivity index (Matsuda index)**	**QUICKI**	**Disposition index**
**Overall**										
Fukushima	5.4 (3.8–8.4)	33.0 (21.1–52.6)	4,445 (3,074–6,622)	0.220 (0.293–0.439)	58.4 (41.7–88.8)	0.73 (0.43–1.22)	1.27 (0.90–1.95)	6.87 (4.82–9.96)	0.369 (0.345–0.391)	80.5 (57.4–149.0)
Nagano	5.9 (4.3–8.1)	38.8 (24.8–55.9)	4,967 (3,501–7,344)	0.248 (0.334–0.487)	70.7 (51.4–93.1)	0.91 (0.57–1.70)	1.40 (0.98–1.95)	6.56 (4.50–8.86)	0.363 (0.345–0.385)	100.0 (61.4–171.9)
Tokushima	4.5 (3.1–6.4)	29.6 (17.9–40.3)	3,670 (2,633–4,925)	0.194 (0.266–0.372)	69.7 (50.6–97.4)	0.69 (0.36–1.12)	1.20 (0.84–1.75)	7.52 (5.65–10.48)	0.372 (0.352–0.395)	82.3 (50.8–128.7)
Okinawa	6.0 (5.0–10.0)	45.0 (32.0–68.0)	6,170 (4,473–8,600)	0.293 (0.401–0.534)	77.2 (55.9–119.3)	0.95 (0.61–1.16)	1.45 (1.01–2.17)	5.57 (3.90–7.49)	0.361 (0.340–0.383)	81.8 (54.2–161.4)
*P* for trend	<0.001	<0.001	<0.001	<0.001	<0.001	<0.001	0.003	<0.001	0.003	0.020
Fukushima*	ns	ns	0.003	0.432	ns	ns	ns	ns	ns	ns
Nagano*	ns	0.008	<0.001	<0.001	ns	<0.001	0.070	0.004	0.080	0.040
Okinawa*	<0.001	<0.001	<0.001	<0.001	0.010	<0.001	0.001	<0.001	0.001	ns
**BMI** **<** **25**										
Fukushima	4.9 (3.4–6.8)	28.0 (19.8–46.6)	4,526 (2,758–6,698)	0.314 (0.182–0.454)	57.0 (31.6–90.2)	0.62 (0.36–1.62)	1.20 (0.82–1.68)	7.30 (4.90–10.75)	0.372 (0.353–0.397)	72.3 (55.0–161.5)
Nagano	4.6 (3.3–6.6)	30.1 (21.1–45.9)	4,143 (3,166–6,000)	0.273 (0.220–0.423)	55.9 (40.7–80.3)	0.73 (0.44–1.58)	1.08 (0.77–1.55)	8.17 (5.72–10.82)	0.379 (0.358–0.401)	101.6 (59.8–179.3)
Tokushima	3.7 (2.7–5.0)	25.5 (16.5–34.2)	3,298 (2,297–4,409)	0.268 (0.206–0.330)	72.0 (55.4–101.4)	0.38 (0.44–1.58)	1.34 (0.91–1.76)	7.37 (5.78–9.06)	0.366 (0.351–0.389)	80.6 (47.9–118.5)
Okinawa	4.7 (3.6–6.8)	36.5 (27.3–58.3)	4,991 (3,584–6,515)	0.346 (0.240–0.439)	64.0 (44.3–90.0)	0.79 (0.46–1.60)	1.04 (0.85–1.47)	7.25 (5.61–9.46)	0.381 (0.360–0.394)	84.1 (58.2–177.0)
*P* for trend	0.030	0.030	<0.001	0.070	0.006	ns	0.070	ns	0.070	ns
Fukushima*	ns	ns	0.010	ns	0.040	ns	ns	ns	ns	ns
Nagano*	ns	ns	<0.001	ns	0.003	ns	0.040	ns	0.040	ns
Okinawa*	ns	0.040	<0.001	0.030	ns	ns	0.070	ns	0.070	ns
**25** **≤** **BMI** **<** **30**										
Fukushima	5.6 (4.0–8.5)	34.5 (22.0–52.5)	4,400 (3,191–6,188)	0.288 (0.233–0.393)	56.4 (45.3–85.0)	0.79 (0.53–1.18)	1.30 (0.92–1.91)	7.00 (4.97–10.14)	0.368 (0.346–0.389)	85.9 (63.4–163.5)
Nagano	6.8 (5.3–9.9)	44.0 (31.5–63.8)	5,387 (3,974–7,588)	0.348 (0.271–0.501)	75.5 (57.6–97.4)	0.99 (0.63–1.65)	1.54 (1.17–2.01)	5.92 (4.37–7.77)	0.358 (0.343–0.374)	98.7 (68.6–177.6)
Tokushima	5.3 (4.6–9.5)	31.0 (19.7–45.8)	3,626 (2,285–5,249)	0.251 (0.178–0.374)	54.0 (44.3–8.5)	0.59 (0.32–1.04)	0.99 (0.62–1.43)	8.53 (6.26–13.22)	0.385 (0.362–0.416)	102.4 (58.6–212.9)
Okinawa	6.6 (4.6–9.5)	47.6 (33.5–69.9)	6,398 (4,472–8,978)	0.408 (0.302–0.568)	80.0 (57.2–118.8)	0.91 (0.56–1.59)	1.55 (1.02–2.18)	5.34 (3.86–7.20)	0.358 (0.339–0.382)	81.3 (52.1–161.6)
*P* for trend	<0.001	<0.001	<0.001	<0.001	<0.001	<0.001	<0.001	<0.001	<0.001	0.070
Fukushima*	0.090	ns	ns	ns	ns	0.040	0.030	ns	0.030	ns
Nagano*	<0.001	0.003	<0.001	<0.001	0.002	<0.001	<0.001	<0.001	<0.001	ns
Okinawa*	<0.001	<0.001	<0.001	<0.001	<0.001	<0.001	<0.001	<0.001	<0.001	ns
**30** **≤** **BMI**										
Fukushima	8.4 (5.0–11.7)	36.5 (39.6–89.8)	4,768 (3,439–8,447)	0.284 (0.227–0.516)	83.9 (45.7–105.6)	0.52 (0.3–1.23)	2.04 (1.17–3.04)	5.22 (3.19–7.84)	0.343 (0.329–0.374)	61.5 (34.8–75.2)
Nagano	11.1 (6.9–17.8)	81.4 (60.0–95.6)	7,589 (6,105–12,081)	0.531 (0.368–0.773)	124.6 (84.0–196.7)	1.41 (0.71–2.50)	2.65 (1.69–4.01)	3.62 (2.34–5.64)	0.330 (0.312–0.353)	97.2 (52.0–127.9)
Tokushima	6.4 (5.2–11.0)	64.3 (43.5–96.8)	4,914 (3,729–9,854)	0.355 (0.246–0.626)	79.2 (51.8–127.8)	0.85 (0.27–1.41)	1.19 (0.78–2.33)	8.58 (3.55–9.31)	0.373 (0.337–0.401)	66.2 (26.4–182.6)
Okinawa	8.8 (6.2–12.4)	75.6 (53.1–96.0)	6,765 (5,540–11,262)	0.472 (0.346–0.637)	110.3 (74.5–153.5)	1.10 (0.79–1.89)	2.17 (1.42–2.84)	4.48 (3.00–5.52)	0.340 (0.327–0.363)	75.7 (52.8–130.2)
*P* for trend	0.020	0.090	0.070	0.030	0.010	0.010	0.020	0.060	0.020	ns
Fukushima*	ns	ns	ns	ns	ns	ns	ns	ns	ns	ns
Nagano*	0.020	ns	ns	ns	0.090	ns	0.009	0.030	0.009	ns
Okinawa*	ns	ns	ns	ns	ns	ns	ns	ns	ns	ns

Data are presented as median (25–75th percentile). BMI, body mass index; AUC, area under the curve; HOMA-β, homeostatic model assessment estimates of β-cell function; HOMA-IR, homeostatic model assessment estimates of insulin resistance; QUICKI, quantitative insulin sensitivity check index; ns, not significant. P-values were obtained by Kruskal–Wallis test, followed by Dunn's multiple comparisons test. P < 0.1 considered statistically significant. * vs. Tokushima.

The region means of anthropometry, biochemical parameters and the daily intake of nutrients and food are shown in Table 2. Ages were different in men (Tokushima > Fukushima > Nagano ≈ Okinawa), but were comparable in women. Among anthropometry and biochemical parameters, BMI, waist circumference, systolic and diastolic blood pressure and triglycerides were higher in Okinawa than in Tokushima and Fukushima and systolic blood pressure (Fukushima > Tokushima) and dietary fiber (Fukushima and Nagano > Tokushima) were different. Characteristics for nutritional indices in four regions were summarized as follows: Fukushima (high intake: grain, fish, carbohydrate calorie, and dietary fiber; low intake: meat and fat calories), Nagano (high intake: grain, fish, and dietary fiber; low intake: meat), Tokushima (at the midpoint in food factors), and Okinawa (high intake: fat calories, low intake: grain, fish, carbohydrate and carbohydrate calories, and dietary fiber).

**Table 2:** The mean values of anthropometry, biochemical parameters and the daily intake of nutrients and food in four regions obtained from the 2016 National Health and Nutrition Survey in Japan.

	**Men**					***P*** **vs. Tokushima**		
**Parameters**	**Fukushima**	**Nagano**	**Tokushima**	**Okinawa**	***P* for trend**	**Fukushima**	**Nagano**	**Okinawa**
Numbers	127	475	100	488				
**Anthropometry and biochemical parameters**								
Age (years)	59.0 (49.0–63.0)	54.0 (45.0–63.0)	61.0 (52.3–65.0)	55.0 (47.0–62.0)	<0.001	ns	<0.001	<0.001
BMI (kg/m^2^)	25.1 (23.5–26.8)	24.8 (23.2– 26.4)	25.2 (23.4–26.8)	26.4 (24.8–28.1)	<0.001	ns	ns	<0.001
Waist circumference (cm)	89.0 (86.5–93.3)	88.0 (84.5–93.0)	88.0 (85.9–93.4)	90.5 (87.0–95.0)	<0.001	ns	ns	0.030
Systolic blood pressure (mmHg)	130.0 (120.0–138.0)	126.0 (116.0–136.0)	124.0 (114.0–136.0)	131.0 (123.3–140.8)	<0.001	0.050	ns	<0.001
Diastolic blood pressure (mmHg)	80.0 (74.0–86.0)	80.0 (69.3–84.0)	78.0 (70.0–84.5)	85.0 (80.0–94.0)	<0.001	ns	ns	<0.001
LDL-cholesterol (mg/dl)	134.0 (113.0–153.0)	130.0 (110.0–148.0)	122.0 (108.5–147.3)	124.0 (106.0–149.4)	0.060	ns	ns	ns
HDL-cholesterol (mg/dl)	51.0 (44.0–59.0)	52.0 (44.0–61.0)	50.0 (43.3–61.0)	48.0 (42.5–58.8)	0.110	ns	ns	ns
Triglyceride (mg/dl)	158.0 (96.0–196.0)	131.0 (88.0–206.0)	140.5 (96.3–211.0)	169.0 (134.3–253.0)	0.003	ns	ns	0.030
**Nutritional indices**								
Energy intake (kcal/day)	2,120 ± 566	2,112 ± 550	2,110 ± 553	1,899 ± 567	<0.001	ns	ns	<0.001
Grain intake (g/day)	522 ± 172	525 ± 170	504 ± 186	437 ± 177	<0.001	ns	ns	<0.001
Meat intake (g/day)	97.9 ± 75.0	104.1 ± 80.3	98.7 ± 78.4	116.0 ± 85.3	0.01	ns	ns	0.007
Fish intake (g/day)	95.0 ± 80.3	84.8 ± 77.3	87.1 ± 80.1	64.1 ± 73.0	<0.001	ns	ns	<0.001
Protein intake (g/day)	78.5 ± 25.9	76.5 ± 23.9	76.9 ± 24.4	70.1 ± 24.4	<0.001	ns	ns	<0.001
Protein calorie (%)	14.9 ± 3.4	14.6 ± 2.8	14.7 ± 4.5	15.0 ± 3.9	ns	ns	ns	ns
Fat intake (g/day)	54.5 ± 33.7	59.3 ± 235	58.3 ± 23.5	62.0 ± 27.8	0.010	ns	ns	ns
Fat calorie (%)	23.5 ± 7.3	25.0 ± 6.9	24.6 ± 7.2	29.1 ± 7.8	<0.001	ns	ns	<0.001
Carbohydrate intake (g/day)	291 ± 83	292 ± 82	289 ± 86	236 ± 75	<0.001	ns	ns	<0.001
Carbohydrate calorie (%)	61.6 ± 9.1	60.4 ± 8.0	60.7 ± 8.3	56.0 ± 9.3	<0.001	ns	ns	<0.001
Dietary fiber (g/day)	16.5 ± 7.5	16.2 ± 7.2	15.1 ± 6.4	13.0 ± 6.6	<0.001	0.020	0.020	<0.001
	**Women**					***P*** **vs. Tokushima**		
**Parameters**	**Fukushima**	**Nagano**	**Tokushima**	**Okinawa**	**P for trend**	**Fukushima**	**Nagano**	**Okinawa**
Numbers	108	434	117	410				
**Anthropometry and biochemical parameters**								
Age (years)	62.0 (58.0–64.0)	59.0 (49.0–64.0)	60.0 (55.0–65.0)	57.0 (50.0–62.0)	<0.001	ns	ns	ns
BMI (kg/m^2^)	26.6 (25.1–28.3)	25.3 (22.9–27.0)	25.0 (22.2–26.1)	26.9 (25.1–28.6)	<0.001	<0.001	ns	<0.001
Waist circumference (cm)	91.0 (86.0–95.0)	88.0 (82.0–93.0)	89.0 (82.8–94.0)	91.8 (88.0–95.8)	<0.001	0.090	ns	<0.001
Systolic blood pressure (mmHg)	132.0 (126.0–141.5)	127.0 (112.0–137.0)	138.0 (114.0–138.0)	132.0 (120.0–140.0)	<0.001	0.050	ns	0.060
Diastolic blood pressure (mmHg)	78.0 (70.3–86.0)	78.0 (70.0–84.0)	74.0 (68.0–82.0)	80.0 (70.0–90.0)	<0.001	0.100	ns	<0.001
LDL-cholesterol (mg/dl)	131.0 (114.0–150.8)	131.0 (111.0–153.0)	136.0 (111.5–155.5)	140.0 (116.8–161.0)	0.020	ns	ns	ns
HDL-cholesterol (mg/dl)	60.0 (49.0–69.0)	60.0 (50.0–70.0)	59.0 (49.0–70.0)	50.0 (41.3–60.0)	<0.001	ns	ns	<0.001
Triglyceride (mg/dl)	99.5 (72.3–161.5)	103.0 (72.3–161.5)	112.0 (72.0–153.0)	166.0 (118.0–.0)	<0.001	ns	ns	<0.001
**Nutritional indices**								
Energy intake (kcal/day)	1,656 ± 492	1,684 ± 412	1,723 ± 421	1,578 ± 465	<0.001	ns	ns	<0.001
Grain intake (g/day)	379.1 ± 132.0	378.0 ± 141.0	371.6 ± 122.6	336.3 ± 135.1	<0.001	ns	ns	<0.001
Meat intake (g/day)	78.4 ± 64.0	72.0 ± 60.0	74.3 ± 59.8	89.4 ± 65.7	<0.001	ns	ns	<0.001
Fish intake (g/day)	82.6 ± 78.0	70.8 ± 61.3	68.3 ± 60.0	52.2 ± 62.0	<0.001	0.006	ns	<0.001
Protein intake (g/day)	65.4 ± 23.6	63.7 ± 18.9	64.5 ± 20.0	60.4 ± 21.7	0.003	ns	ns	0.002
Proitein calorie (%)	15.8 ± 3.2	15.2 ± 3.0	15.1 ± 4.6	15.4 ± 3.6	0.080	0.040	ns	ns
Fat intake (g/day)	48.3 ± 20.7	50.5 ± 19.9	51.3 ± 20.4	54.5 ± 20.8	0.020	ns	ns	ns
Fat calorie (%)	26.0 ± 7.0	26.7 ± 7.3	26.5 ± 7.5	30.2 ± 7.5	<0.001	ns	ns	<0.001
Carbohydrate intake (g/day)	232 ± 74	235 ± 64	244 ± 66	204 ± 64	<0.001	0.030	0.030	<0.001
Carbohydrate calorie (%)	58.2 ± 8.7	58.1 ± 8.3	58.4 ± 8.5	54.4 ± 8.9	<0.001	ns	ns	<0.001
Dietary fiber (g/day)	15.4 ± 7.4	15.5 ± 0.7	15.3 ± 6.9	13.2 ± 6.7	<0.001	ns	ns	<0.001

Data are presented as mean ± SD or median (25–75th percentile). BMI: body mass index. P-values were obtained by Kruskal–Wallis test, followed by Dunn's multiple comparisons test. P < 0.1 considered statistically significant. * vs. Tokushima.

**Figure 2:**
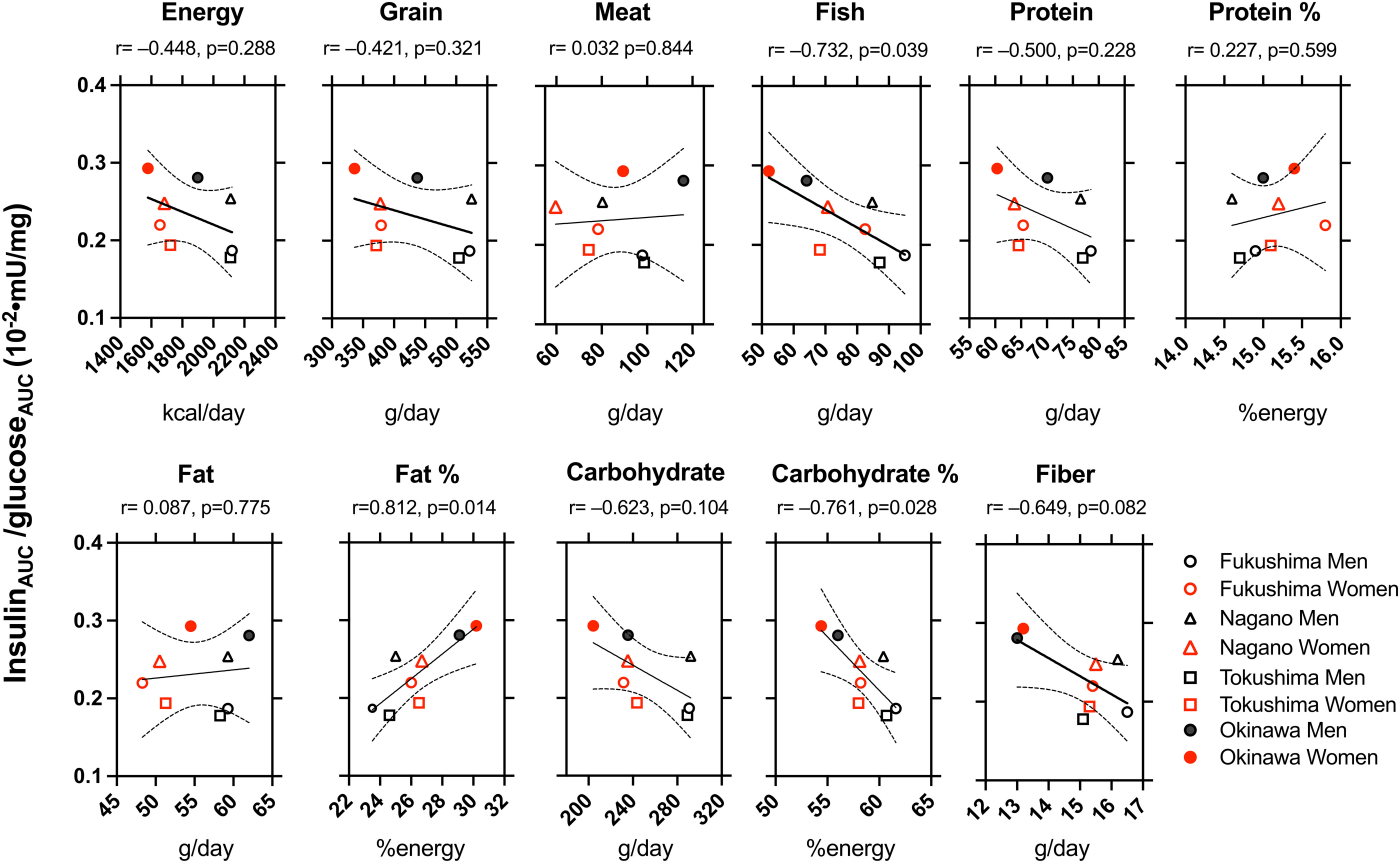
The correlation between the indices of dietary factors and indices of insulin secretion and sensitivity in Fukushima men (°) and women (°), Nagano men (Δ) and women (Δ), Tokushima men (□) and women (□), and Okinawa men (•) and women (•). *r*: The Pearson product correlation coefficient and *p*: probability. Solid and dotted lines indicate r with 95% confidential intervals.

The correlation between the indices of nutrients and food intake (Table 3) and insulin secretion and sensitivity in four regions are shown in Supplementary Figure 6 and Figure 2. Overall, insulin_120_ and insulin_AUC_/glucose_AUC_ correlated negatively with fish intake, carbohydrate intake, and carbohydrate calories and positively correlated with fat calories. However, HOMA-β was negatively correlated with fish intake and carbohydrate calories and positively correlated with fat calories.

**Table 3:** Simple and multiple regression analysis to estimate insulin_AUC_/glucose_AUC_.

	**Model 1 (Unadjusted)**		**Model 2 (Age)**		**Model 3 (Sex)**		**Model 4 (BMI)**	
**Parameters**	**β**	***P***	**β**	***P***	**β**	***P***	**β**	***P***
Energy intake (kcal/day)	−0.448	0.265	−0.682	0.011	−2.112	0.046	−0.144	0.770
Grain intake (g/day)	−0.421	0.299	−0.662	0.017	−1.564	0.133	−0.114	0.814
Meat intake (g/day)	0.032	0.939	−0.148	0.684	0.330	0.603	−0.143	0.720
Fish intake (g/day)	−0.732	0.039	−0.607	0.038	−0.885	0.048	−0.604	0.160
Protein intake (g/day)	−0.500	0.207	−0.649	0.016	−1.988	0.026	−0.250	0.593
Protein calorie (%)	0.227	0.589	0.715	0.021	0.194	0.797	−0.419	0.445
Fat intake (g/day)	0.087	0.215	−0.543	0.192	1.702	0.088	0.199	0.606
Fat calorie (%)	0.812	0.014	0.677	0.014	0.891	0.023	0.877	0.076
Carbohydrate intake (g/day)	−0.623	0.099	−0.694	0.004	−1.164	0.049	−0.453	0.455
Carbohydrate calorie (%)	−0.761	0.028	−0.664	0.011	−0.934	0.032	−0.815	0.143
Dietary fiber (g/day)	−0.649	0.082	−0.538	0.088	0.102	0.775	0.189	0.730

*β, standardized partial regression coefficient; P, provability. P < 0.1 considered statistically significant*.

**Figure 3:**
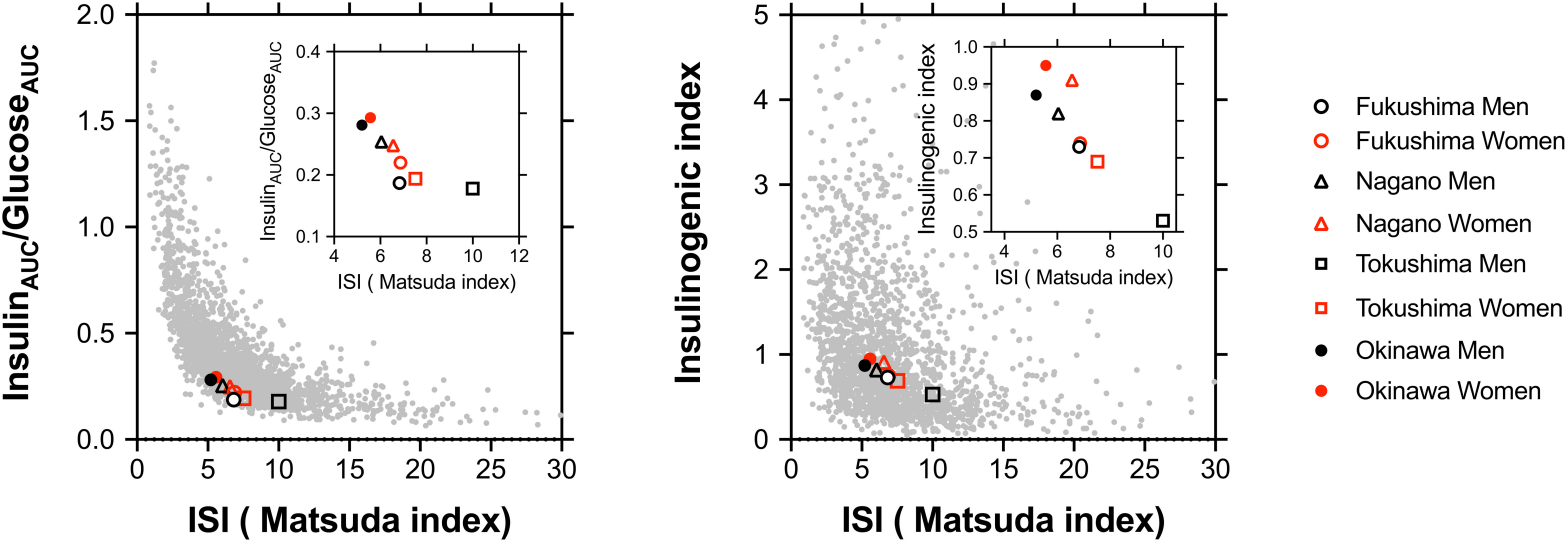
The plot between the insulin sensitivity index (ISI; Matsuda index) and insulin_AUC_/glucose_AUC_ (left panel) or the insulinogenic index (right panel) in all participants including those from four regions. The stabilization points of insulin sensitivity and the insulin response are depicted on plots in all participants and are shown in the inset as Fukushima men (°) and women (°), Nagano men (Δ) and women (Δ), Tokushima men (□) and women (□), and Okinawa men (•) and women (•).

As shown in Figure 3, the plot between the insulin sensitivity index (Matsuda index) and insulin_AUC_/glucose_AUC_ (left panel) or the insulinogenic index (right panel) for all participants showed hyperbolic relationships. In men or women, as shown in the figure insets, the points shifted from the lower right toward the upper left in the following order: Tokushima < Fukushima < Nagano < Okinawa.

Finally, we performed a multiple regression analysis to estimate insulin_AUC_/glucose_AUC_ (Table 3). After corrected for age (Model 2) or sex (Model 3), energy intake, fish intake, protein intake, fat calorie and carbohydrate intake and calorie were significantly associated with insulin_AUC_/glucose_AUC_. However, after corrected for BMI (Model 4), only fat calorie showed a borderline significance.

## Discussion

We compared the indices of insulin response and sensitivity of men and women with NGT in four different areas of Japan, and noted two major findings. First, there were regional variations in insulin secretion and sensitivity. This difference was observed even within the same BMI category. Second, the indices of insulin secretion correlated positively with fat intake and negatively with the intake of fish, carbohydrate calories, and dietary fiber. Although racial/ethnic differences in the optimal balance for insulin secretion and insulin sensitivity are well-known, this study is the first to report that the optimal balance differs significantly among participants from different regions in Japan and that nutritional factors could be linked to these differences, independently of body size/adiposity. Furthermore, this study suggests that the insulin response and insulin sensitivity can vary among adult individuals, even within the same race and the same country, and is therefore likely affected by environmental/lifestyle factors independently of genetic traits.

### Regional Variations in Indices of Insulin Secretion and Insulin Sensitivity

The insulin secretory capacity and insulin sensitivity differ between races. Kodama et al. discovered that the stabilization points of insulin sensitivity and insulin response in a hyperbolic relationship model differ among African, Caucasian, and East Asian NGT cohorts (2). For healthy NGT subjects, differences in body size/compositions are well-known among Africans (less visceral fat and more skeletal muscle mass than Caucasians), Caucasians (more visceral fat and less skeletal muscle mass than Africans), and East Asians (less visceral fat and smaller height, weight, and BMI than the other two ethnic groups). Therefore, it is possible that the differences in insulin secretion and sensitivity may be affected by differences in body size/composition. Another explanation for this difference is the innate capacity for insulin secretion, namely genetic susceptibility (22, 23). A recent meta-analysis of genome-wide association studies in 77,418 East Asians with type 2 diabetes mellitus revealed that East Asians share common variants for type 2 diabetes mellitus with Europeans, but also exhibit unique undescribed associations of those variants (24). In this study, we selected only Japanese participants, and examined the relationship between dietary factors and indices of insulin secretion and sensitivity in participants from distinct regions. We found significant regional differences in insulin response and sensitivity, even within participants of the same race and country: the order of the insulin responses was Okinawa > Nagano > Fukushima > Tokushima (Figure 1A).

As obesity/adiposity might have an impact on insulin secretion and sensitivity, we subdivided the participants according to their BMI. In men, the insulin responses were Nagano ≈ Okinawa > Fukushima ≈ Tokushima in BMI <25 and Fukushima ≈ Nagano ≈ Okinawa > Tokushima in 25 ≤ BMI <30 and BMI ≥ 30 (Figure 1A). In women, the insulin responses were Nagano ≈ Okinawa > Fukushima ≈ Tokushima in BMI <25 and 25 ≤ BMI <30 (Figure 1B). Thus, regional differences in insulin response were assumed partially due to body size/adiposity, but also due to other unrecognized factors. The larger variability [median (25–75th percentile)] of insulin levels in men from Fukushima, Nagano and Okinawa may suggest an intra-regional heterogeneity. We assessed representative indices for insulin secretion and insulin sensitivity proposed (17–19, 21) and found interregional difference in some indices (Insulin_AUC_, Insulin_AUC_/Glucose_AUC_, HOMA-β, insulinogenic index, HOMA-IR, Matsuda index, and QUICKI), but not in some (Disposition index). Although we cannot explain the discrepancy from our results, several possibilities need to be considered such as difference in diagnostic quality and/or mechanistic background which might be affectete by different foods or nutrients, i.e., fish, fat, carbohydrate in these indices.

Racial/ethnic differences in the optimal balance for insulin secretion and insulin sensitivity are dependent on body size/adiposity and/or genetic background (2). We found that the specified points of the insulin sensitivity index (Matsuda index) and insulin secretion (insulin_AUC_/glucose_AUC_) were clearly different for the four regions, and these variations cannot be explained only by the degree of obesity and visceral fat given the variations in the differences in the distribution of insulin response among the four regions even after correction for BMI.

### Potential Mechanisms: Dietary Factors and Insulin Secretion

We evaluated the correlation between dietary factors and indices of insulin secretory capacity and sensitivity in men and women from the four Japanese regions. Insulin_120_, insulin_AUC_/glucose_AUC_, and HOMA-β were negatively correlated with the intake of fish, carbohydrate calories, and dietary fiber and were positively correlated with fat calories (Figure 2 and Supplementary Figure 6). Three possible mechanisms might explain the regional difference in insulin response as differences in the intake of: fatty acids; carbohydrate, and fish.

### Difference in SFA Intake

In this present study, the indices of insulin secretion did not correlate with the daily energy intake, but positively correlated with fat-derived energy and negatively with carbohydrate calories and dietary fiber. In the multiple regression analysis to estimate insulin_AUC_/glucose_AUC_, only fat calorie showed a borderline significance after corrected for BMI (Table 3). Marshall et al. reported that fasting insulin levels were positively correlated with fat intake and negatively associated with carbohydrate intake in Hispanic and Caucasian cohorts from Colorado, USA (25). A report in the Netherlands reported that fasting C-reactive peptide was associated positively with the total fat intake and negatively with carbohydrates (17). Maron et al. reported that total fat and SFA intakes positively and negatively correlated with fasting insulin secretion and carbohydrate intake, respectively, in non-diabetic individuals with coronary artery disease (CAD) (26). In contrast to the abovementioned studies (17, 25, 26) that evaluated such relationships in fixed regions, our study compared the average values in multiple regions, suggesting that the geographical differences in the insulin response may be linked to the degree of the intake of fat, carbohydrate, and dietary fiber.

Insulin secretion was augmented in Japanese immigrants in Seattle, USA (3, 22) and Hawaii, USA (27) as compared to that in native Japanese. In Pima Indians in the USA, the transition from an agrarian to a modern society was associated with the consumption of increasing amounts of dietary fat, decreasing amounts of dietary carbohydrate, and a decline in insulin sensitivity (28). In Japan, the era of westernization is fairly different among the four regions tested. In the earlier era (c.1945–1972) after World War II, the American food culture under the presence of US military bases in Okinawa caused a rapid shift in food culture from the Japanese (29) to a westernized dietary style (30, 31). In contrast, the other three regions more gradually shifted from the Japanese to a westernized dietary style for topographic, historical, and cultural reasons (12, 29, 32).

This study did not conclusively ascertain the reason why fat calories are related to insulin secretion, but one can presume two potential mechanisms. First, the SFA in the high-fat diet may cause insulin resistance. Second, the SFA may directly alter insulin secretion. Previously, we reported that SFA stimulates insulin secretion in normal animal models independently of glucose concentration (8, 9). McGarry et al. have shown that FFA directly stimulates insulin secretion in cultured pancreatic β-cells by glucose-insulin coupling, and that the response is affected by chain length and the FFA degree of saturation (7). Incretins are hormones secreted from the digestive tract on nutrient intake that act on pancreatic β-cells (33). Gastric inhibitory polypeptide (GIP), but not glucagon-like peptide-1 (GLP-1), can be stimulated by dietary fat in individuals with NGT (34). Currently, it is unknown whether postprandial incretin secretion is linked to the relationship between fat intake and insulin response. Taken above all, insulin sensitivity and insulin secretion are mutually and closely interlinked, and FFA effects might modulate the stabilization points of insulin sensitivity and insulin response (1, 21).

### Difference in Carbohydrate Intake

The fasting insulin level correlated negatively with carbohydrate intake in healthy Hispanic and White cohorts (25), normal healthy Netherlanders (17), and Americans with coronary artery disease (26). Insulin levels positively correlated with sucrose intake and negatively correlated with complex carbohydrates and dietary fiber (25). Our results agree with previous studies (17, 25, 26). Low carbohydrate intake supposedly lowers insulin levels, increases circulating FFA levels, and enhances fatty acid oxidation (25, 26). There is a possibility that insulin sensitivity and insulin response may differ by the type of carbohydrates. Diets with high dietary fiber intake show a low glycemic index, wherein even the same amount of calories can reduce postprandial hyperinsulinemia, easily reduce body fat, and improve insulin sensitivity (35). The differences in carbohydrate intake in the four regions tested may reflect the differences in habits of consuming grains and fats (12, 29–32). Because there was no difference in protein intake among the four regions, there is a possibility that a difference in low carbohydrate calories and high fat calories may be linked to differences in insulin secretion. Low carbohydrate calories can reduce the circulating insulin level, which promotes higher circulating FFA levels for use in oxidation and production of ketone bodies (36). Therefore, it would be interesting to examine whether the difference in the glycemic index is linked to regional differences in insulin secretion.

### Difference in Fish Intake

We found that fish intake was associated with decreased insulin secretion. The intake of meat (beef and chicken), when matched for macronutrient and energy content, is more insulinogenic than that of fish (37). Insulin secretion may be reduced because of improved sensitivity from a higher fish intake (38). Conversely, fish n-3 PUFA may play a role in transcriptional regulation for adipocytokines and protection of the insulin secretory capacity through membrane receptors (39). The four regions of Japan differed greatly in the intake ratio of meat and fish, which may cause changes in fatty acid composition (40), thereby affecting insulin secretion and sensitivity (37–39).

### Other Potential Mechanisms: Discrepancies Between the Different Indexes and Interindividual Variability of the Indexes for Insulin Secretion and Sensitivity

As discussed above, we found interregional difference in some indices, but not in some, used to measure insulin secretion and sensitivity. We have no biological explanation for the discrepancies between the different indexes. The notion may be also considered that these discrepancies, together with the large interindividual variability in the results and in the cardiometabolic phenotypic characteristics of the participants could result in false interregional differences. True mechanisms for “interregional difference” need to be clarified in future studies which will investigate possible determinants for insulin secretion and sensitivity including more precise individual characteristics.

### Strengths and Limitations

The strengths of this study are the use of nationally representative data on dietary intake in Japan that were obtained by a standardized and unified protocol for population-level surveys. Moreover, as we used the large database of national health insurance in Japan, we could compare the relationship between dietary factors and indices of insulin response and sensitivity in the general population. However, this study has several limitations. First, we compared the dietary factors and OGTT-based indices of insulin secretion and sensitivity from different datasets and combined the data into representative variables for the four regions. Therefore, the results obtained from the two datasets should be interpreted cautiously. The correlation between dietary factors and variables observed in this study need to be confirmed by future studies using the same dataset for the relationship between dietary intake and insulin (secretion/sensitivity) indices. Second, we selected four regions for comparison, but the selection may be biased and, perhaps, non-representative of the nationwide data. Third, the utility of the household-based dietary record in estimating energy and nutrient intake is limited and the household response rates for the 2016 NHNS were low (44.4% in 2016) (12), which possibly could cause bias. Fourth, measurements of OGTT were optional at routine annual specific health checkups, and this is another possible source of bias. Fifth, the intra-regional variability for potential confounding factors such as age, BMI and other metabolic alterations may be relevant for the differences in the insulin response and insulin sensitivity. However, we did not perform multivariate analysis including these potential confounders because limited numbers for nutritional indices (*n* = 8) may cause overfitting multiple regression models such as misleading coefficients. Therefore, we limited explanatory parameters to two in the models (Table 3).

## Conclusion

This is the first study to evaluate regional differences in insulin response and insulin sensitivity in Japanese participants and to show significant regional differences in these indices and that nutritional factors may be linked to these differences independently of body size/adiposity. Furthermore, insulin response and insulin sensitivity can vary among adult individuals, even within the same race and the same country, and are therefore likely affected by environmental/lifestyle factors independently of genetic traits.

## Data Availability Statement

The raw data supporting the conclusions of this article will be made available by the authors, without undue reservation.

## Ethics Statement

The studies involving human participants were reviewed and approved by the Fukushima Medical University Ethics Committee (approval number 30168). An opt-out notice for tacit consent was posted to publish the research information and ensure the opportunity for refusal. The ethics committee waived the requirement of written informed consent for participation.

## Author Contributions

KW analyzed data and wrote first manuscript. MSh designed the study, analyzed data, and wrote the manuscript with input from all authors. MH, YH, AK, RA, BW, DW, MSa, and HM contributed to the discussion and edited the manuscript. MSh is the guarantor of this work and, as such, had full access to all the data in the study and takes responsibility for the integrity of the data and the accuracy of the data analysis. All authors contributed to the article and approved the submitted version.

## Conflict of Interest

The authors declare that the research was conducted in the absence of any commercial or financial relationships that could be construed as a potential conflict of interest.
