# Supplementary figures and images for: Regional Variations of Insulin Secretion and Insulin Sensitivity in Japanese Participants With Normal Glucose Tolerance
Source: Front Nutr. 2021 Mar 22;8:632422. doi: 10.3389/fnut.2021.632422 (PMC8019818; doi:10.3389/fnut.2021.632422)

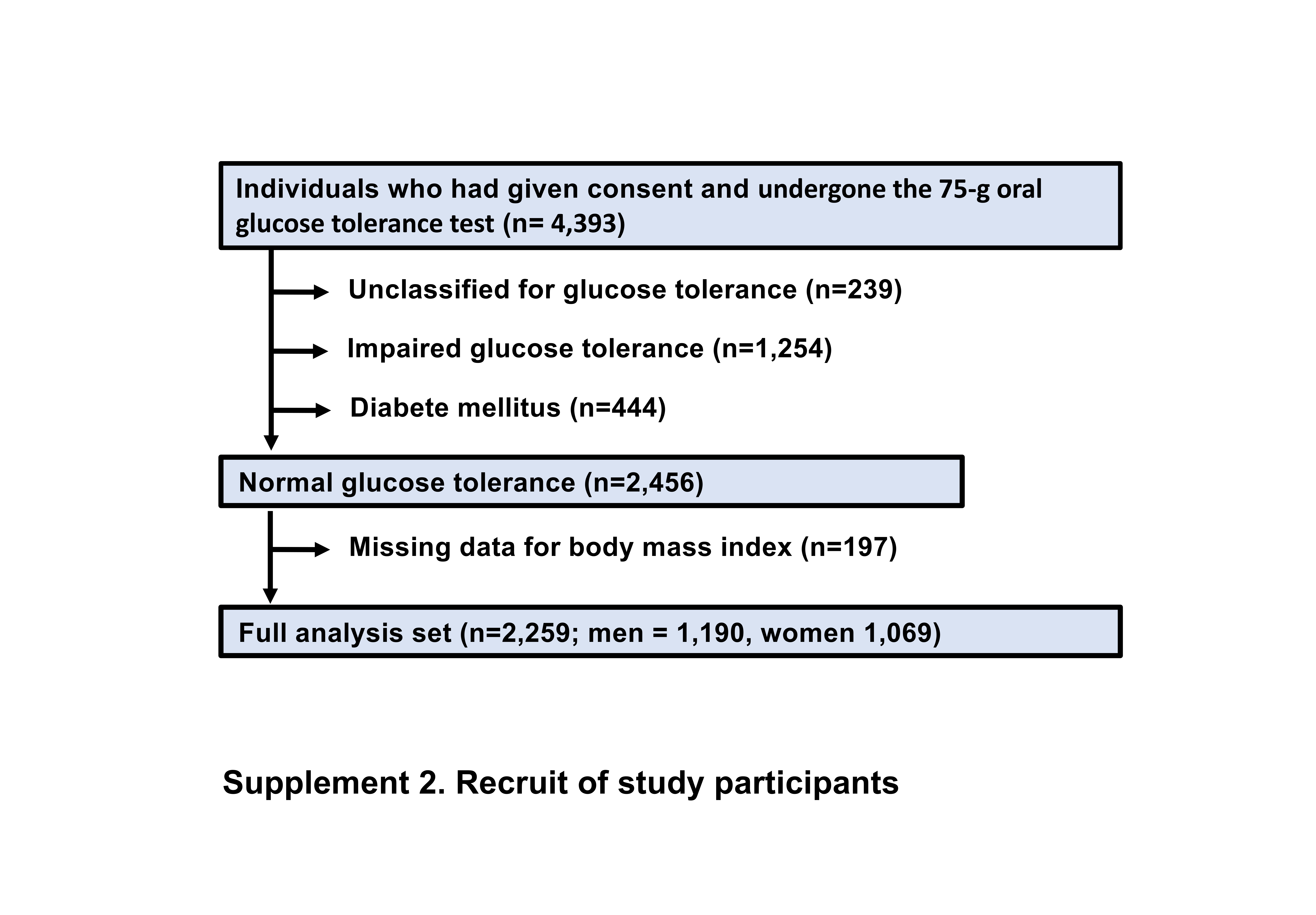

Supplement: Supplementary file 2 [file Image_2.tif]
