# Supplementary material for: Regional Variations of Insulin Secretion and Insulin Sensitivity in Japanese Participants With Normal Glucose Tolerance
Source: Front Nutr. 2021 Mar 22;8:632422. doi: 10.3389/fnut.2021.632422 (PMC8019818; doi:10.3389/fnut.2021.632422)
Supplement: Supplementary file 4 [file Table_1.pdf]

Supplement 3. Baseline characteristics of participants

Men

BMI <25

						vs Tokushima		
Variables	Fukushima	Nagano	Tokushima	Okinawa	P	Fukushima	Nagano	Okinawa
n	60	252	47	137				
Age, years	60.0 [52.0 - 63.0]	57.0 [48.0-63.0]	59.5 [53.0 - 63.0]	57.0 [50.0-62.0]	<0.001	ns	<0.001	0.010
BMI, kg/m^2	23.4 [22.1 - 24.4]	23.3 [21.8 - 24.1]	23.3 [22.3 - 24.4]	23.9 [22.9 - 24.6]	<0.001	ns	ns	ns
(min-max)	(18.8 - 24.9)	(17.9 - 24.9)	(19.6 - 24.9)	(19.3 - 24.9)				
Waist circumference, cm	87.3 [85.0 - 89.0]	85.3 [80.8 - 87.9]	86.0 [81.2 - 87.5]	86.5 [84.0 - 88.0]	0.001	ns	ns	ns
Systolic blood pressure, mmHg	131.0 [120.0 - 138.0]	124.0 [114.0-133.0]	134.0 [112.0 -134.0]	130.0 [120.0 - 140.0]	<0.001	0.070	ns	0.020
Diastolic blood pressure, mmHg	79.0 [74.0 - 84.0]	78.0 [71.0-85.0]	78.0 [70.0 - 84.0]	83.0 [76.0 - 93.0]	<0.001	ns	ns	<0.001
LDL-cholesterol, mg/dl	131.5 [110.8 - 149.0]	129.0 [112.0-147.0]	123.5 [111.3 - 150.8]	121.0 [105.0 - 148.8]	0.320	ns	ns	ns
HDL-cholesterol, mg/dl	51.0 [45.0-60.8]	54.0 [47.0-63.0]	49.5 [44.3 - 61.0]	49.0 [44.0 - 62.0]	0.230	ns	ns	ns
Triglyceride, mg/dl	118.0 [82.0 - 186.0]	133.0 [75.0-229.5]	165.0 [135.0 - 280.0]	129.5 [85.0 -196.3]	0.030	ns	ns	ns

25≤ BMI <30

						vs Tokushima		
Variables	Fukushima	Nagano	Tokushima	Okinawa	P	Fukushima	Nagano	Okinawa
n	57	201	48	288				
Age, years	58.0 [50.0 - 62.0]	56.0 [46.0-63.0]	60.0 [50.0 - 64.8]	56.0 [47.0 - 63.0]	0.230	ns	ns	ns
BMI, kg/m^2	26.4 [25.7 - 27.8]	26.3 [25.7 - 27.5]	26.5 [25.6 - 27.5]	26.8 [25.9 - 27.8]	0.050	ns	ns	ns
(min-max)	(25.0 - 29.9)	(25.0 - 29.9)	(25.1 - 29.9)	(25.0 - 29.9)				
Waist circumference, cm	91.0 [89.0 - 95.0]	91.8 [89.0 - 96.0]	92.0 [89.0 - 96.0]	91.2 [89.0 - 95.0]	0.880	ns	ns	ns
Systolic blood pressure, mmHg	134.0 [124.0 - 139.0]	128.0 [118.0-138.0]	130.0 [115.0 -140.0]	132.0 [126.0 - 142.0]	<0.001	ns	ns	ns
Diastolic blood pressure, mmHg	79.0 [74.0 - 84.0]	78.0 [71.0-85.0]	78.0 [70.0 - 84.0]	83.0 [76.0 - 93.0]	<0.001	ns	ns	0.020
LDL-cholesterol, mg/dl	131.5 [110.8 - 149.0]	129.0 [112.0-147.0]	123.5 [111.3 - 150.8]	121.0 [105.0 - 148.8]	0.300	ns	ns	ns
HDL-cholesterol, mg/dl	51.0 [45.0-60.8]	54.0 [47.0-63.0]	49.5 [44.3 - 61.0]	49.0 [44.0 - 62.0]	0.650	ns	ns	ns
Triglyceride, mg/dl	118.0 [82.0 - 186.0]	133.0 [75.0-229.5]	165.0 [135.0 - 280.0]	129.5 [85.0 -196.3]	0.130	ns	ns	ns

30≤ BMI

						vs Tokushima		
Variables	Fukushima	Nagano	Tokushima	Okinawa	P	Fukushima	Nagano	Okinawa
n	10	22	5	63				
Age, years	48.0 [43.8 - 62.3]	46.0 [35.5 - 51.3]	63.0 [50.0 - 67.0]	50.0 [42.0 - 61.0]	0.080	ns	0.040	ns
BMI, kg/m^2	32.2 [30.7 - 34.7]	32.4 [30.7 - 34.7]	32.5 [31.3 - 35.1]	31.4 [30.6 - 33.0]	0.320	ns	ns	ns
(min-max)	(30. 1- 37.7)	(30.1 - 37.6)	(31.2 - 36.3)	(30.0 - 39.5)				
Waist circumference, cm	103.8 [100.2 - 109.9]	103.8 [100.0 - 110.0]	105.0 [98.7 - 106.5]	103.0 [95.3 - 108.0]	0.490	ns	ns	ns
Systolic blood pressure, mmHg	121.0 [117.5 - 133.0]	135.0 [120.5 - 142.3]	116.0 [114.0 - 130.0]	130.0 [124.0 - 142.0]	0.070	ns	ns	ns
Diastolic blood pressure, mmHg	81.5 [76.5 - 87.0]	86.0 [78.3 - 90.0]	76.0 [65.0 - 82.0]	90.0 [80.0 - 110.0]	0.005	ns	ns	0.020
LDL-cholesterol, mg/dl	131.5 [110.8 - 149.0]	129.0 [112.0-157.0]	110.0 [77.0 - 111.0]	135.0 [111.0 - 148.8]	0.060	ns	ns	ns
HDL-cholesterol, mg/dl	40.0 [35.8 - 48.3]	44.0 [38.5 - 56.0]	47.0 [35.0 - 67.0]	45.5 [39.5 - 54.5]	0.670	ns	ns	ns
Triglyceride, mg/dl	161.0 [82.0 - 186.0]	152.0 [125.0 - 279.3]	107.0 [70.0 - 212.0]	172.0 [124.3 - 214.8]	0.580	ns	ns	ns

Women

BMI <25

						P vs Tokushima		
Variables	Fukushima	Nagano	Tokushima	Okinawa	P for trend	Fukushima	Nagano	Okinawa
n	26	190	59	86				
Age, years	62.0 [60.0 - 64.3]	59.0 [46.8-64.0]	59.0 [48.0 - 66.0]	59.5 [55.0-63.0]	0.020	0.050	ns	ns
BMI, kg/m^2	23.4 [21.7 - 24.5]	22.6 [20.1 - 23.9]	22.3 [20.7 - 23.9]	23.4 [21.7 - 24.5]	0.010	ns	ns	0.040
(min-max)	(17.7 - 24.9)	(16.7 - 24.9)	(17.3 - 24.9)	(16.7 - 24.9)				
Waist circumference, cm	90.0 [80.5 - 91.9]	81.1 [74.5 - 88.5]	84.5 [75.8 - 89.6]	86.8 [89.5 - 91.0]	<0.001	ns	ns	ns
Systolic blood pressure, mmHg	133.0 [123.5 - 146.0]	121.0 [107.0 - 134.0]	122.0 [110.0 -136.0]	120.0 [111.0 - 138.3]	0.006	0.040	ns	ns
Diastolic blood pressure, mmHg	80.0 [72.0 - 88.0]	80.0 [74.0-88.0]	70.0 [62.0 - 80.0]	82.0 [78.0 - 92.0]	0.170	ns	ns	ns
LDL-cholesterol, mg/dl	132.0 [114.0-150.3]	132.0 [109.0-153.0]	133.5 [108.5 - 155.3]	136.0 [114.8 - 159.3]	0.680	ns	ns	ns
HDL-cholesterol, mg/dl	63.5 [47.0 - 74.5]	65.0 [52.0-74.0]	55.0 [47.5 - 67.0]	50.0 [47.8 - 68.3]	<0.001	ns	0.060	0.020
Triglyceride, mg/dl	97.5 [73.5 - 134.8]	85.0 [64.0-137.5]	130.5 [72.3 - 170.3]	173.0 [125.8 - 249.8]	<0.001	ns	ns	<0.001

25≤ BMI <30

						P vs Tokushima		
Variables	Fukushima	Nagano	Tokushima	Okinawa	P for trend	Fukushima	Nagano	Okinawa
n	68	212	50	267				
Age, years	62.0 [56.0 - 64.0]	59.0 [52.0 - 64.0]	61.0 [55.8 - 65.0]	59.0 [54.0 - 63.0]	0.030	ns	ns	0.060
BMI, kg/m^2	26.8 [25.9 - 28.1]	26.4 [25.9 - 28.1]	25.9 [25.4 - 26.7]	27.0 [26.1 - 28.1]	<0.001	0.005	ns	<0.001
(min-max)	(25.0 - 29.9)	(25.0 - 29.9)	(25.0 - 29.9)	(25.0 - 29.9)				
Waist circumference, cm	90.9 [86.6 - 94.2]	90.5 [87.0 - 94.9]	91.5 [87.0 - 94.8]	92.0 [89.0 - 95.0]	0.080	ns	ns	ns
Systolic blood pressure, mmHg	130.0 [125.3 - 138.0]	130.0 [119.0-140.0]	130.0 [120.0 -138.0]	134.0 [122.0 - 142.0]	0.020	ns	ns	ns
Diastolic blood pressure, mmHg	79.0 [74.0 - 84.0]	78.0 [71.0-85.0]	78.0 [70.0 - 84.0]	83.0 [76.0 - 93.0]	0.001	ns	ns	0.004
LDL-cholesterol, mg/dl	130.0 [110.5 - 151.8]	130.0 [111.0-151.3]	141.0 [112.0 - 161.0]	141.0 [117.3 - 161.0]	0.050	ns	ns	ns
HDL-cholesterol, mg/dl	60.0 [49.0 - 69.0]	58.0 [49.0 - 65.6]	66.0 [53.0 - 77.5]	49.0 [41.0 - 60.0]	<0.001	ns	0.080	<0.001
Triglyceride, mg/dl	121.0 [72.3 - 171.3]	113.0 [78.8-157.0]	112.0 [79.5 - 140.0]	163.5 [121.0 -236.0]	<0.001	ns	ns	<0.001

30≤ BMI

						P vs Tokushima		
Variables	Fukushima	Nagano	Tokushima	Okinawa	P for trend	Fukushima	Nagano	Okinawa
n	14	32	8	57				
Age, years	62.0 [58.8 - 64.5]	55.0 [41.0 - 61.0]	61.5 [55.5 - 63.8]	57.0 [51.5 - 59.0]	0.006	ns	ns	ns
BMI, kg/m^2	31.7 [30.6 - 32.5]	31.6 [30.5 - 33.9]	31.5 [31.0 - 34.8]	33.0 [31.6 - 35.0]	0.040	ns	ns	ns
(min-max)	(30.0 - 39.1)	(30.5 - 37.9)	(30.9 - 36.8)	(30.0 - 41.6)				
Waist circumference, cm	101.6 [97.3 - 105.2]	102.0 [98.8 - 105.5]	101.0 [95.1 - 107.9]	101.0 [93.5 - 107.2]	0.820	ns	ns	ns
Systolic blood pressure, mmHg	136.0 [130.0 - 142.5]	130.0 [114.5 - 141.5]	154.0 [137.0 - 162.0]	136.0 [130.0 - 142.0]	0.040	ns	0.020	ns
Diastolic blood pressure, mmHg	85.5 [78.5 - 88.5]	80.0 [72.3 - 87.8]	91.0 [81.5 - 97.3]	82.0 [79.0 - 98.0]	0.040	ns	ns	ns
LDL-cholesterol, mg/dl	131.0 [122.0 - 140.0]	130.5 [117.3 - 150.8]	136.5 [71.8 - 156.0]	134.0 [115.3 - 164.0]	0.930	ns	ns	ns
HDL-cholesterol, mg/dl	55.0 [45.8 - 62.0]	51.5 [43.3 - 65.3]	58.5 [48.5 - 65.0]	52.5 [44.3 - 60.0]	0.610	ns	ns	ns
Triglyceride, mg/dl	97.0 [63.8 - 136.0]	142.0 [88.5 - 215.5]	62.5 [49.0 - 100.5]	172.0 [109.8 - 233.8]	<0.001	ns	0.020	0.001

Data are presented as median [25 - 75th percentile]. BMI: body mass index, LDL: low density lipoprotein; HDL: high density lipoprotein. P values were obtained by Kruskal–Wallis test, followed by Dunn's multiple comparisons test.
