# Supplementary material for: Regional Variations of Insulin Secretion and Insulin Sensitivity in Japanese Participants With Normal Glucose Tolerance
Source: Front Nutr. 2021 Mar 22;8:632422. doi: 10.3389/fnut.2021.632422 (PMC8019818; doi:10.3389/fnut.2021.632422)
Supplement: Supplementary file 5 [file Table_2.pdf]

Supplement 4. Oral glucose tolerance test

Men

Glucose (mg/dL)

Overall

	0	30	60	120
Fukushima	96 [92 - 101]	154 [142 - 171]	144 [124 - 172]	110 [98 - 120]
Nagano	96 [91 - 101]	150 [133 - 168]	141 [115 - 169]	107 [92 - 121]
Tokushima	95 [89 - 100]	157 [135 - 175]	150 [123 - 179]	107 [92 - 122]
Okinawa	97 [92 - 102]	159 [138 - 178]	149 [129 - 180]	116 [99 - 127]

BMI<25

	0	30	60	120
Fukushima	97 [91 - 102]	153 [136 - 174]	145 [124 - 170]	110 [94 - 121]
Nagano	95 [90 - 100]	150 [134 - 167]	137 [111 - 166]	104 [89 - 120]
Tokushima	91 [84 - 99]	144 [101 - 158]	127 [101 - 158]	108 [98 - 123]
Okinawa	97 [91 - 101]	155 [134 - 176]	143 [122 - 169]	113 [95 - 126]

25≤BMI<30

	0	30	60	120
Fukushima	96 [93 - 102]	157 [144 - 171]	147 [123 - 177]	110 [100 - 120]
Nagano	96 [91 - 102]	148 [132 - 170]	137[117 - 169]	109 [96 - 122]
Tokushima	90 [85 - 98]	138 [115 - 162]	124 [101 - 148]	107 [95 - 119]
Okinawa	97[93 - 103]	162 [140 - 181]	154 [133 - 181]	117 [102 - 129]

BMI ≤ 30

	0	30	60	120
Fukushima	93 [98 - 96]	144 [130 - 163]	144 [129 - 180]	114 [100- 120]
Nagano	94 [89 - 98]	156 [137 - 175]	159 [125 - 178]	112 [93 - 121]
Tokushima	76 [73 - 79]	125 [111 - 129]	96 [83 - 130]	93 [87 - 128]
Okinawa	95 [89 - 102]	153 [89 - 102]	158 [126 - 182]	117 [95 - 125]

Insulin (mU/L)

Overall

	0	30	60	120
Fukushima	5.5 [4.2 - 7.9]	42.7 [27.5 - 68.7]	51.2 [31.8 - 80.9]	26.7 [17.7 - 47.5]
Nagano	5.9 [4.2-8.0]	50.2 [31.2 - 79.7]	57.6 [39.1 - 89.6]	34.5 [21.8 - 55.6]
Tokushima	4.4 [3.0 - 6.2]	34.3 [20.0 - 51.3]	41.8 [28.0 - 63.7]	25.4 [14.5 - 39.7]
Okinawa	7.0 [5.0 - 10.0]	58.0 [39.0 - 90.0]	71.0 [44.0-112.0]	43.0 [25.0 - 69.0]

BMI<25

	0	30	60	120
Fukushima	4.3 [3.2 - 5.8]	32.6 [21.0 - 49.7]	41.7 [29.1 - 58.8]	22.0 [19.8 - 46.6]
Nagano	5.0 [3.5 - 6.7]	45.2 [26.8 - 67.6]	49.5 [32.1 - 49.8]	29.3 [19.8 - 46.6]
Tokushima	3.4 [2.6 - 5.1]	31.1 [16.7 - 48.6]	38.2 [21.4 - 48.9]	21.9 [12.2 - 32.9]
Okinawa	5.0 [4.1 - 6.4]	45.8 [30.5 - 78.3]	56.8 [44.0-112.0]	34.1 [18.9 - 49.8]

25≤BMI<30

	0	30	60	120
Fukushima	7.1 [4.9 - 9.4]	57.5 [38.0 - 80.9]	58.8 [38.9 - 92.0]	33.9 [21.8 - 55.8]
Nagano	5.0 [3.5 - 6.7]	58.7 [40.0 - 90.0]	71.3 [48.3 - 118.1]	42.4 [25.2 - 66.0]
Tokushima	4.8 [3.8 - 6.5]	38.5 [23.9 - 59.1]	52.8 [29.7 - 80.6]	25.8 [14.2 - 43.6]
Okinawa	7.0 [4.9 - 9.5]	55.6 [39.7 - 88.8]	70.2 [44.1-109.3]	44.2 [26.8 - 73.0]

BMI≥30

	0	30	60	120
Fukushima	7.6 [3.9 - 9.7]	43.5 [36.1 - 76.3]	59.2 [33.4 - 118.0]	45.3 [14.8 - 98.6]
Nagano	6.8 [5.3 - 9,9]	54.0 [39.2 - 77.5]	54.4 [36.8 - 83.2]	44.4 [31.5 - 63.8]
Tokushima	5.3 [3.8 - 6.8]	34.4 [23.9 - 56.2]	39.0 [23.6 - 54.0]	31.0 [19.7 - 45.8]
Okinawa	7.0 [4.9 - 9.5]	55.6 [39.7 - 88.8]	70.2 [44.1-109.3]	44.2 [26.8 - 73.0]

Women

Glucose (mg/dL)

Overall

	0	30	60	120
Fukushima	97 [93 - 101]	149 [141 - 168]	135 [107 - 159]	113 [100 - 125]
Nagano	94 [90 - 99]	143 [125 - 159]	130 [105 - 154]	114 [98 - 124]
Tokushima	91 [85 - 98]	144 [122 - 162]	129 [105 - 155]	110 [97 - 129]
Okinawa	93 [88 - 98]	151 [129 - 167]	136 [117 - 164]	116 [107 - 127]

BMI<25

	0	30	60	120
Fukushima	98 [94 - 103]	161 [126 - 177]	136 [98 - 188]	108 [92 - 126]
Nagano	93 [89 - 99]	140 [124 - 161]	123 [102 - 154]	110 [92 - 124]
Tokushima	94 [87 - 100]	145 [133 - 162]	134 [109 - 163]	113 [93 - 125]
Okinawa	93 [88 - 97]	147 [128 - 169]	126 [128 - 169]	118 [105 - 127]

25≤BMI<30

	0	30	60	120
Fukushima	96 [92 - 99]	143 [125 - 158]	128 [107 - 149]	111 [100 - 124]
Nagano	94 [90 - 99]	146 [127 - 159]	132 [106 - 153]	115 [101 - 124]
Tokushima	90 [84 - 97]	138 [118 - 160]	122 [103 - 148]	111 [103 - 124]
Okinawa	93 [88 - 99]	152 [119 - 166]	136 [119 - 166]	116 [104 - 129]

BMI ≤ 30

	0	30	60	120
Fukushima	103 [96 - 106]	164 [148 - 178]	159 [141 - 165]	121 [112 - 135]
Nagano	96 [91 - 100]	143 [129 - 163]	141 [112 - 162]	117 [109 -126]
Tokushima	92 [83 - 95]	150 [126 - 199]	150 [113 - 179]	114 [107 - 129]
Okinawa	94 [90 - 99]	154 [137 - 167]	154 [119 - 178]	119 [107 - 125]

Insulin (mU/L)

Overall

	0	30	60	120
Fukushima	5.4 [3.8 - 8.4]	40.3 [28.2 - 58.8]	43.3 [31.1 - 63.4]	33.0 [21.1 - 52.6]
Nagano	5.9 [4.3 - 8.1]	47.4 [32.2 - 70.2]	47.7 [33.0 - 72.4]	38.8 [24.8 - 55.9]
Tokushima	4.5 [3.1 - 6.4]	31.8 [22.5 - 51.4]	35.6 [21.5 - 53.1]	29.6 [17.9 - 40.3]
Okinawa	6.0 [5.0 - 10.0]	56.0 [37.0 - 87.0]	60.0 [41.0 - 92.5]	45.0 [32.0 - 68.0]

BMI<25

	0	30	60	120
Fukushima	4.9 [3.4 - 6.8]	39.3 [25.3 - 66.0]	41.2 [28.3 - 64.9]	28.0 [19.8 - 46.6]
Nagano	4.6 [3.3 - 6.6]	40.0 [26.5 - 58.9]	41.7 [27.9 - 62.4]	30.1 [21.1 - 45.9]
Tokushima	3.7 [2.7 - 5.0]	28.7 [20.6 - 48.7]	30.1 [20.5 - 48.7]	25.5 [16.5 - 34.2]
Okinawa	4.7 [3.6 - 6.8]	48.7 [30.4 - 82.9]	46.1 [33.5 - 66.4]	36.5 [27.3 - 58.3]

25≤BMI<30

	0	30	60	120
Fukushima	5.6 [4.0 - 8.5]	403 [38.3 - 57.4]	43.6 [31.4 - 62.5]	34.5 [22.0 - 52.5]
Nagano	6.8 [5.3 - 9.9]	54.0 [39.2 - 77.5]	54.4 [23.9 - 56.2]	44.0 [31.5 - 63.8]
Tokushima	5.3 [4.6 - 9.5]	34.4 [23.9 - 56.2]	39.0 [23.6 - 54.0]	31.0 [19.7 - 45.8]
Okinawa	6.6 [4.6 - 9.5]	55.0 [37.9 - 90.4]	63.4 [43.8 -97.4]	47.6 [33.5 - 69.9]

BMI≥30

	0	30	60	120
Fukushima	8.4 [5.0 - 11.7]	36.5 [39.6 - 89.8]	48.2 [43.5 - 96.3]	49.7 [26.7 - 69.0]
Nagano	11.1 [6.9 - 17.8]	81.4 [60.0 - 95.6]	71.9 [52.4 - 119.7]	69.9 [45.1 - 119.7]
Tokushima	6.4 [5.2 - 11.0]	64.3 [43.5 - 96.8]	61.2 [40.6 - 116.1]	47.1 [39.1 - 90.7]
Okinawa	8.8 [6.2 - 12.4]	75.6 [53.1 - 96.0]	73.2 [53.1 - 138.3]	54.4 [34.2 - 77.3]

Data are presented as median [25 - 75th percentile]. BMI: body mass index
